# PhyloFacts: an online structural phylogenomic encyclopedia for protein functional and structural classification

**DOI:** 10.1186/gb-2006-7-9-r83

**Published:** 2006-09-14

**Authors:** Nandini Krishnamurthy, Duncan P Brown, Dan Kirshner, Kimmen Sjölander

**Affiliations:** 1 Department of Bioengineering, 473 Evans Hall #1762, University of California, Berkeley, CA 94720, USA

## Abstract

The Berkeley Phylogenomics Group presents PhyloFacts, a structural phylogenomic encyclopedia containing almost 10,000 'books' for protein families and domains, with pre-calculated structural, functional and evolutionary analyses. PhyloFacts enables biologists to avoid the systematic errors associated with function prediction by homology through the integration of a variety of experimental data and bioinformatics methods in an evolutionary framework. Users can submit sequences for classification to families and functional subfamilies. PhyloFacts is available as a worldwide web resource from .

## Abstract 2

PhyloFacts, a structural phylogenomic database for protein functional and structural classification, is described.

## Rationale

Computational methods for protein function prediction have been critical in the post-genome era in the functional annotation of literally millions of novel sequences. The standard protocol for sequence functional annotation - transferring the annotation of a database hit to a sequence 'query' based on predicted homology - has been shown to be prone to systematic error [1-3]. The top hit in a sequence database may have a different function to the query due to neofunctionalization stemming from gene duplication [4], differences in domain structure [5,6], mutations at key functional positions, or speciation [1]. Annotation errors have been shown to propagate through databases by the application of homology-based annotation transfer [7-9]. While the exact frequency of annotation error is unknown (one published estimate is 8% or higher [7]), the importance of detecting and correcting existing errors and preventing future errors is undisputed.

An additional complicating factor in annotation transfer by homology is the complete failure of this approach for an average of 30% of the genes in most genomes sequenced: in some cases no homologs can be detected within a particular significance threshold, for instance, a BLAST [10] expectation (E) value (that is, the number of hits receiving a given score expected by chance alone in the database searched) of 0.001 or less, while in other cases database hits may be labeled as 'hypothetical' or 'unknown'.

With the huge array of bioinformatics software tools and resources available, it might seem unthinkable that functional annotation accuracy would be so difficult to ensure. Rather like the parable of the blind men and the elephant, each tool used separately provides a partial and imperfect picture; taken as a whole, the probable molecular function of the protein, biological process, cellular component, interacting partners, and other aspects of a protein's function can often come into better focus. For instance, annotation transfer from the top BLAST hit may suggest a protein is a receptor-like protein kinase, while domain structure prediction reveals that no kinase domain is present; the two orthogonal analyses prevent mis-annotation of the unknown protein.

In this paper we present PhyloFacts, an online structural phylogenomics encyclopedia containing almost 10,000 'books' for protein families and domains, designed to improve the accuracy and specificity of protein function prediction [11]. PhyloFacts integrates a wide array of biological data and informatics methods for protein families, organized on the basis of structural similarity and by evolutionary relationships. This enables a biologist to examine a rich array of experimental data and bioinformatics predictions for a protein family, and to quickly and accurately infer the function of a protein in an evolutionary context.

## Annotation accuracy requires data and method integration

PhyloFacts is motivated by two of the biggest lessons of the post-genome era - the power of integrating data and inference tools from different sources, and improved prediction accuracy using consensus approaches in bioinformatics. For instance, protein structure prediction 'meta-servers' making predictions based on a consensus over results retrieved from several independent servers typically have lower error rates than any one server used separately [12]. In the case of protein structure prediction, we can also take advantage of the fact that members of a large diverse protein family tend to share the same three-dimensional structure even when their primary sequence similarity becomes undetectable. This enables us to use another type of consensus approach involving the application of the same method to several different members of the family to boost prediction accuracy (for example, [13]).

We employ the same basic principles in this resource, by integrating many different prediction methods and sources of experimental data over an evolutionary tree. In cases where attributes are known to persist over long evolutionary distances (such as protein three-dimensional structure), we can integrate predictions over the entire tree to derive a consensus prediction for the family as a whole. In cases where attributes are more restricted in their distribution in the family (for example, ligand recognition among G-protein coupled receptors), inferences will be more circumspect, potentially restricted to strict orthologs. Evolutionary and structural clustering of proteins enables us to integrate these disparate types of data and inference methods effectively, to identify potential errors in database annotations and provide a platform to improve the accuracy of functional annotation overall.

In addition to new methods developed by us for phylogenomic inference, PhyloFacts includes a number of standard bioinformatics methods available publicly. To motivate the need for protein functional classification integrating diverse methods and data in an evolutionary framework, we examine the major classes of bioinformatics methods in turn, and discuss their different pros and cons. Methods designed for predicting the biological process(es) in which a protein participates (for example, bioinformatics approaches such as Phylogenetic Profiles [14] and Rosetta Stone [15], analysis of DNA chip array data, and proteomics experiments such as pull-down experiments, yeast two-hybrid data, and so on) are clearly complementary, and will be included in future releases of the PhyloFacts resource.

### Database homolog search tools

Database homolog search tools (for example, BLAST, FASTA [16], and so on) can be blindingly fast, but do not distinguish between local matches and sequences sharing global similarity; they report a score or E-value measuring the significance of the local match between a query sequence and sequences in the database. This can lead to errors when annotations are transferred *in toto *based on only local similarity. These pairwise sequence comparison methods of homolog detection have also been shown to have limited effectiveness at recognizing remote homologs (distantly related sequences) [17].

### Iterated homology search methods

Iterated homology search methods such as PSI-BLAST [10] have been developed in recent years. These methods enable larger numbers of sequences to be annotated functionally, albeit with a potentially higher error rate due to divergence in function from their common ancestor.

### Domain-based annotation and protein structure prediction

Domain-based annotation and protein structure prediction libraries of profiles or hidden Markov models (HMMs) for functional or structural domains (PFAM [18], SMART [19], or Superfamily [20]) are particularly helpful when a homolog search fails. There are two primary limitations of this approach to functional annotation. First, these statistical models of protein families and domains are typically designed for sensitivity rather than specificity, and thus afford a fairly coarse level of annotation. For example, the PFAM 7TM_1 HMM recognizes a variety of G-protein coupled receptors, irrespective of their ligand specificity. Second, a protein's function is a composite of all its constituent domains; thus, even in cases where each of a protein's domains can be identified, the actual function of the protein may not be elucidated.

### Phylogenomic inference

Phylogenomic inference was originally designed to address the problem of annotation transfer from paralogous rather than orthologous genes through the construction and analysis of phylogenetic trees overlaid with experimental data. This approach has been shown to enable the highest accuracy in prediction of protein molecular function [21-23], but inherent technical and computational complexity has limited its use. Several attempts at identification of orthologs (for example, Orthostrapper [24] and RIO [25]) and at automating phylogenomic inference of molecular function [26] have been presented, and may lead to more widespread application of this approach.

### Prediction of protein localization

Prediction of protein localization is enabled by resources such as the TMHMM [27] transmembrane prediction server, the TargetP [28] cellular component prediction server, and the PHOBIUS [29] integrated signal peptide and transmembrane prediction server. These provide another perspective on a protein's function, and can suggest participation in biological pathways when other data are lacking. Because these methods can rely on fairly weak and non-specific signals (for example, hydrophobic stretches as indicators of membrane localization), both false positive and false negative predictions are not uncommon [30].

## The PhyloFacts phylogenomic encyclopedia

As of 11 July 2006, the PhyloFacts encyclopedia contains 9,710 'books' for protein superfamilies and structural domains. Each book in the PhyloFacts resource contains heterogeneous data for protein families, including a cluster of homologous proteins, multiple sequence alignment, one or more phylogenetic trees, predicted three-dimensional structures, predicted functional subfamilies, taxonomic distributions, Gene Ontology (GO) annotations [31], PFAM domains, hyperlinks to key literature and other online resources, and annotations provided by biologist experts. Residues conferring family and subfamily specificity are predicted using alignment/evolutionary analyses; these patterns are plotted on three-dimensional structures. HMMs constructed for each family and subfamily enable classification of novel sequences to different functional classes. Details on each aspect of the resource construction are available in the 'Details on Library Construction and Software Tools' section.

Slightly more than half of the books in the PhyloFacts resource represent experimentally determined structural domains; the remaining fraction is divided between global homology groups (GHGs: globally alignable proteins having the same domain structure), conserved regions, motifs, and 'Pending', a label for those books that have not passed the stringent requirements for global homology and must be manually examined. Each book is labeled with the book type ('domain', 'global homology', and so on) to enable appropriate functional inferences. These labels are based primarily on multiple sequence alignment analysis. See Table 1 for the number of books within each class.

The PhyloFacts phylogenomic resource can be used in several ways: sequences can be submitted for protein structure prediction or functional classification, protein family books can be browsed, and data of various types (multiple sequence alignments (MSAs), phylogenetic trees, HMMs, and so on) can be downloaded from the resource.

### Browsing PhyloFacts

**Figure 1:**
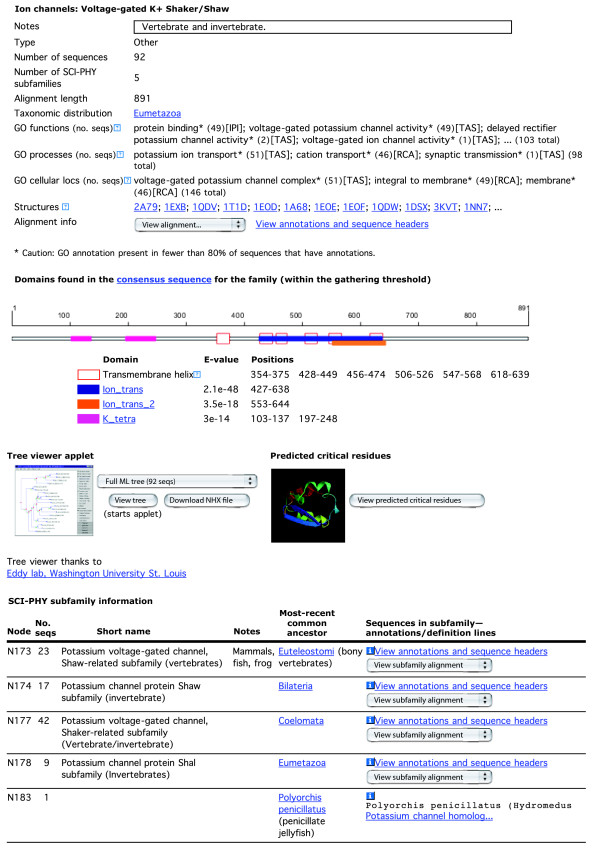
PhyloFacts book: Voltage-gated K+ channels, Shaker/Shaw subtypes. Each book contains summary data at the top of the book page, including book type, number of sequences, number of predicted subfamilies, and taxonomic distribution. PFAM domains matching the book consensus sequence are displayed along with predicted transmembrane domains and signal peptides. Phylogenetic trees and multiple sequence alignments can be viewed or downloaded, for the family as a whole or for individual subfamilies. Predicted critical residues have been identified and are plotted on homologous PDB structures, where available (Figure 5). Clicking on 'View annotations and sequence headers' displays GO annotations and evidence codes for sequences in the family as a whole and for individual subfamilies.

Each of the books in the library has a corresponding web page [32] for viewing the associated annotation and experimental data, MSA, trees, predicted domain structures, and so on (Figure 1).

### Sequence analysis

**Figure 2:**
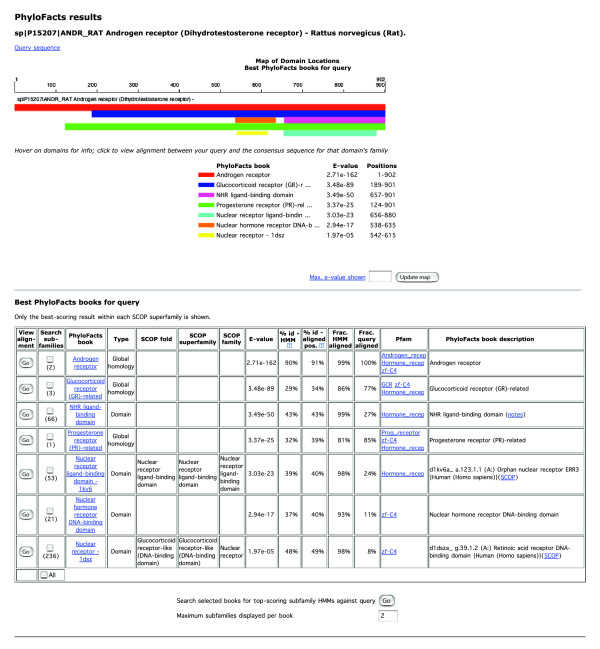
PhyloFacts search results for ANDR_RAT, androgen receptor from *Rattus norvegicus*. Books with significant scores are displayed graphically at top, followed by various statistics about each match in a table below. The top-scoring book (red bar) represents a global homology group of Androgen receptors, which matches the entire query sequence. Examining the table below shows the Androgen receptor book has an E-value of 2.71e-162, 91% identity between the query and book consensus (based on aligned residues), and high fractional coverage of the HMM (99%). Other global homology groups retrieved include evolutionarily related Glucocorticoid and Progesterone receptors, but analysis of query coverage and percent identity shows the Androgen receptor book to provide a superior basis for annotation transfer. Other books displayed include structural domains detected in the query. Two books (for the ligand-binding domain 1kv6a and the DNA-binding domain 1dsza) were constructed for the Structure Prediction series based on SCOP domains. Subsequent construction of the specialized book series on transmembrane receptors in the human genome resulted in additional books being constructed for these domains. Scoring subfamily HMMs is enabled by selecting the 'Search subfamilies' box (second column in the spreadsheet of results, shown checked in the figure), and clicking on the 'Go' button at bottom ('Search selected books for top-scoring subfamily HMMs against query'). Clicking on the 'Go' button below 'View alignment' in the first column brings up a separate page displaying the pairwise alignment of the query and the family consensus sequence along with relevant statistics about the alignment. Clicking on the hyperlink to the book itself (in the 'PhyloFacts book' column) retrieves the webpage for the family (see example book page shown in Figure 1).

Classification to a protein family is enabled by HMM scoring. Biologists can submit either nucleotide or amino acid sequences in FASTA format; nucleotide sequences are first translated into all six frames and analyzed separately. Batch mode submission of up to five sequences is enabled. Results are returned by e-mail, and allow users to select families for more detailed classification of sequences to functional subfamilies based on scoring against subfamily HMMs (Figure 2). This functionality is available online [33].

PhyloFacts includes books focusing on specific protein families or classes. The largest of these series is the PhyloFacts 'Protein Structure Prediction' library, with 5,328 books, each representing either a structural domain from the Astral database [34] or protein structures from the Protein Data Bank (PDB [35]). This series enables biologists to obtain predicted structures for submitted proteins. The books in the Protein Structure Prediction library were created using individual structural domains as seeds, gathering homologs from the NR [36] database using PSI-BLAST or the UCSC SAM [37] software tools.

The second major book series in PhyloFacts is the 'Animal Proteome Explorer' library, containing 4,226 protein families in the human genome, expanded to include additional homologs from other organisms. Specialized sections of the Animal Proteome Explorer series are devoted to protein families of particular biomedical relevance: G-Protein Coupled Receptors (65 books), Ion Channels (50 books), and Innate Immunity (52 books). The Animal Proteome Explorer series has been constructed using GHGCluster (see section 'Details on Library Construction and Software Tools'). The GPCR library includes books for protein families based on the classification of the GPCRDB [38].

The 'Plant Disease Resistance Phylogenomic Explorer' forms the third main series of specialized books in PhyloFacts, devoted to protein families involved in plant disease resistance and host-pathogen interaction (105 books). Families in this series include the canonical plant *R *(resistance) genes, proteins involved in defense signaling and effector proteins from plant pathogens.

These three main divisions are not strictly distinct, and there are some overlaps. For instance, a book for the Toll Interleukin Receptor (TIR) domain (PhyloFacts book ID: bpg002615) is placed in the Protein Structure Prediction library (due to the presence of a solved structure for this family) as well as in the Innate Immunity and Plant Disease Resistance libraries (since TIR domains are found in both plant and animal proteins involved in eukaryotic innate immunity).

Because our recommended protocol for protein function prediction starts with transfer of annotation from globally alignable orthologs (see section 'Functional annotation using PhyloFacts'), a large number of books in PhyloFacts are designated as type Global Homology, and subjected to rigorous quality control (see section 'Details on Library Construction and Software Tools, Defining Book Type'). Standard protein clustering tools typically ignore the issue of global sequence similarity, so that even resources intending to cluster proteins based on global similarity can occasionally fail (for example, the Celera Panther resource [39] class Leucine-Rich Transmembrane Proteins [PTHR23154] contains proteins with diverse domain structures; Additional data file 1). By contrast, most web servers for protein functional classification provide primarily domain-level analyses (for example, SMART and PFAM). To supplement these analyses, PhyloFacts also provides books for different types of structural similarities across sequences, including short conserved motifs and structural domains.

PhyloFacts has other distinguishing features relative to other online resources. In contrast to model organism databases that are restricted to a single species (for example [40-43]) sequences in PhyloFacts are clustered into protein families with potentially diverse phylogenetic distributions, enabling biologists to benefit from experimental studies in related species. GO annotations and evidence codes are provided for each subfamily separately as well as for the family as a whole. Phylogenetic trees are constructed for each protein family, using Neighbor-Joining, Maximum Likelihood and Maximum Parsimony methods. Analysis of the full phylogenetic tree topology, along with GO annotations and evidence codes, allows biologists to avoid the systematic errors associated with annotation transfer from top database hit. Protein structure prediction and domain analysis are presented to enable biologists to take advantage of the unique information provided by protein structure studies. Simultaneous evolutionary and structural analyses enable us to predict enzyme active sites and other types of key functional residues. HMMs for each family and subfamily provide functional classification of user-submitted sequences at different levels of a functional hierarchy. This enables functional annotation that can be far more specific than what is provided by typical protein family or domain classification web servers. A detailed comparison of PhyloFacts with some of the standard functional classification servers is presented in Table 2.

PhyloFacts currently includes almost 10,000 books providing pre-calculated phylogenomic analyses for protein superfamilies and structural domains, and over 700,000 HMMs enabling classification of user-submitted sequences to families and subfamilies. Between 64% and 82% of genes encoded in different model organism genomes can be classified at least at the domain level to one or more books in the PhyloFacts resource (Table 3). PhyloFacts coverage is constantly increasing. We have currently completed clustering and expansion of the human genome, resulting in 10,163 global homology group clusters. Of these, approximately 3,969 clusters (representing 38% of human genes) have been installed in the PhyloFacts resource (although not all of them have passed the stringent GHG requirements); remaining books are in various stages of completeness.

## Functional annotation using PhyloFacts

In an ideal scenario, annotation transfer between a query and homolog would meet three criteria [22]: first, global homology; second, orthology [44]; and third, supporting experimental evidence for the functional annotation being transferred. In practice, confirming agreement at all three criteria is not always straightforward. Very few sequences have experimentally solved structures; satisfaction of the first condition is, therefore, typically determined by comparison of their predicted domain structures using, for example, PFAM or Conserved Domain [45] analysis, or by pairwise alignment analysis. Automated determination of orthology is complicated due to incomplete sequencing, gene duplication and loss, errors in gene structure and other issues; for a review see [46]. Satisfying the last condition is equally difficult due to the paucity of sequences with experimentally determined function; our analysis of GO annotations and evidence codes for over 370,000 sequences in the UniProt database [47] shows <3% to have experimental evidence supporting a functional annotation. (This statistic is based on the analysis of 372,448 UniProt sequences present in the PhyloFacts resource as of June 2005. Two-thirds of these (248,152) had GO annotations, but only 3% of this smaller set had evidence codes indicating experimental support: IDA (inferred from direct assay), IGI (inferred from genetic interaction), IMP (inferred from mutant phenotype), IPI (inferred from physical interaction), and TAS (traceable author statement).)

Books in the PhyloFacts resource are labeled by the level of structural similarity across members (that is, global homology, domain, and so on), and include phylogenetic trees, inferred subfamilies, and GO annotations and evidence codes to enable a biologist to check for agreement at the three criteria for transferring annotations. In cases where a protein of unknown function is placed in a global homology group with an ortholog having experimentally determined function, annotation transfer can proceed with high confidence. In other cases, the biologist can check for experimentally determined function in paralogous genes (bearing in mind that functions may have diverged), or at domain-based clusters, to obtain clues to the molecular function for different regions of a protein of interest. We attempt to accommodate all of these possibilities; a sequence search against the resource may match books representing global homology groups, structural domains, conserved regions, or even short motifs, all of which are presented to the user (Figure 2).

We note that while domain-based annotation is inherently less precise, PhyloFacts does provide predicted functional subfamilies within domain-based books as well as within books representing global homology groups. While annotation transfer across proteins having different overall folds is prone to systematic error, previous results suggest that subfamily classification of sequences aligned along a single common domain can be consistent with the overall domain structure and molecular function of sequences [48]. Our experiments using SCI-PHY to analyze proteins with different overall domain structures also support the same conclusion (unpublished data, Brown DP, Krishnamurthy N, Sjölander K).

In addition to the value PhyloFacts presents to a human investigator, it also provides a framework for the development of a fully automated functional inference system. A new generation of probabilistic methods for inferring molecular function automatically has arisen in recent years (for example, [26,49,50]). For instance, SIFTER uses a Bayesian approach to infer a distribution over possible functions in a phylogenetic tree, taking as input a cluster of sequences, a phylogenetic tree, and GO annotations and evidence codes, all of which PhyloFacts collects and integrates in one resource. SIFTER integration is to be available in our next release.

However, technical issues present barriers to the goal of fully automated function prediction (see [51] for a review). Sequences in a cluster may have different descriptors based on the species of origin; for example, the *Drosophila *community is likely to use different names for a gene to that used by the *Caenorhabditis elegans *community, and both are likely to use different terms to those used by investigators working in mouse genomics. The value of a standardized nomenclature, such as that being developed by GO, is obviously important, but significant work remains in this area. An exhaustive thesaurus of equivalent biological terms would be valuable. The sparse nature of experimentally supported molecular functions provides an additional barrier to automated approaches. We discuss these issues further in the section 'Challenges to phylogenomic inference'.

Clustering together proteins based on predictable global homology enables us to analyze a cluster of homologs as a unit and detect potential errors in annotation; database annotation errors tend to stand out as anomalous against a backdrop of otherwise consistent annotations (unless, of course, annotation errors have percolated through the database).

For instance, the *Oryza sativa *GenBank protein AAR00644 is labeled as a 'putative LRR receptor-like protein kinase'. The canonical structure of receptor-like kinases (RLKs) consists of an extracellular leucine-rich repeat (LRR) region, a transmembrane domain, and a cytoplasmic kinase domain; AAR00644 contains no kinase domain. On the other hand, AAR00644 does match the canonical structure of closely related receptor-like proteins (RLPs), which are structurally very similar to RLKs, except that they terminate with a short cytoplasmic tail, and do not contain a kinase domain [52]. In the PhyloFacts resource, this protein is classified as a member of the global homology group book 'Plant LRR proteins (putative RLPs)' (PhyloFacts book ID: bpg005632), where PFAM domain analysis of the cluster shows no detectable kinase domains.

For a second example, the GenBank sequence AAF19052 labeled as 'neutral human sphingomyelinase' [53] appears to be neither human nor a sphingomyelinase. Instead it appears to encode a bacterial isochorismate synthase protein. This sequence is classified to the PhyloFacts book 'Isochorismate synthase-related' (Phylofacts book ID: bpg004927), in which this purportedly 'human' sequence is the only representative eukaryote. (Note that even the translated BLAST search of this sequence against the human genome finds no matches.) In this case, both domain structure analysis and analysis of the taxonomic distribution of the globally homologous members of the family help identify the probable error.

Lastly, G-protein coupled receptor (GPCR) classification is notoriously difficult, with many receptors having no known ligand (termed 'orphan receptors'). One such orphan, a GPCR from river lamprey (UniProt: Q9YHY4), is annotated as 'Putative odorant receptor LOR3', based on its expression in the olfactory epithelium [54]. Standard profile/HMM-based analyses (for example, PFAM, SMART and the NCBI CDD) only match this protein to the PFAM 7TM_1 class, containing dozens of subtypes. BLAST analysis shows other putative odorant receptors from river lamprey (submitted by the same authors) as top hits, followed by trace amine receptors. However, analyses of phylogenetic trees containing this sequence show it (and the other putative odorant receptors detected by BLAST) to be located within subtrees containing trace amine receptors (see PhyloFacts books bpg004950, bpg000525 and bpg000543) and to be quite different from experimentally confirmed odorant receptors (Additional data file 2).

Anomalous annotations such as these are often signs that annotation transfer has gone wrong. In other cases, anomalies may be quite real and provide new insights into the evolution of novel functions in a family. Automated anomaly detection faces the same technical barriers as automated functional annotation, including the need for probabilistic inference of gene function, standardized nomenclatures and exhaustive synonym tables of biological terms. At present, these anomalies - whether true functional differences or database annotation errors - are detected manually. In the future we expect automated function prediction methods will enable anomalous annotations to be flagged for expert examination. Protocols will then need to be established by the biological community to correct any errors and to ensure that sequence databases receive corrected annotations.

## Details on resource construction and software tools

**Figure 3:**
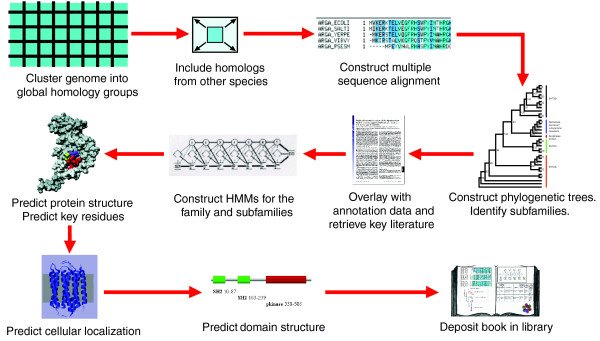
PhyloFacts whole-genome library construction pipeline. This figure represents our protocol for building global homology group protein family books. The pipeline starts with clustering a target genome into global homology groups (GHGs; sequences sharing the same overall domain structure), and proceeding through various stages of cluster expansion, multiple sequence alignment, phylogenetic tree construction, retrieval of experimental data, a variety of bioinformatics methods for predicting functional subfamilies, key residues, cellular localization, and so on, and quality control assessment.

Construction of the PhyloFacts resource required the development of a computational pipeline (shown in Figure 3), software for classifying user-submitted sequences, and graphical user interfaces. These are outlined briefly below.

### Clustering sequences for PhyloFacts books

Sequences for structural domain books were gathered using PSI-BLAST and UCSC SAM Target-2K (T2K) [37]. Sequences retrieved for global homology group books are required to share the same overall domain structure (global alignment). We have two tools for this process: FlowerPower (NK, Brown D, KS, unpublished data) and GHGCluster.

#### FlowerPower

FlowerPower is an iterative homolog detection algorithm like PSI-BLAST that retrieves homologs to a seed sequence (or query) and aligns sequences using profile methods. However, instead of using a single profile to identify and align new sequences, FlowerPower uses subfamily identification and subfamily HMM construction to expand the homology cluster in each iteration. Alignment analysis is used to restrict the cluster to match user-specified criteria (for example, global alignment for protein function prediction using phylogenomic inference, and global-local alignment (global to the seed, local to the database hit) for domain-based clustering). Experimental validation of FlowerPower shows it has greater selectivity than BLAST, PSI-BLAST and the UCSC SAM-T2K methods of homolog detection at discriminating sequences with local similarity from those with global similarity. The FlowerPower server is available online [55].

#### GHGCluster

The Global Homology Group (GHG) Cluster program enables us to cluster a selected sequence database (for example, a genome) into global homology groups, while also including homologs from a second, generally larger, database.

GHGCluster takes two inputs: a set of sequences *Q*, containing the sequences to be clustered, and a database *D *to use for expanding the clusters to include globally alignable homologs from other organisms. A superset of sequences, the expansion database *E*, is created by merging *Q *and *D*. To improve run time, *E *is partitioned into overlapping bins based on sequence length. A seed sequence (query) is chosen from *Q *and homologs are gathered from its corresponding bin in *E*, using PSI-BLAST (E-value < 1e-5; user-specified number of iterations). Each hit is assessed for global homology to the query, based on percent identity (≥20%), and bi-directional alignment coverage, that is the fractional aligned length of both seed and hit (ranging from 60% for sequences <100 residues to 85% for sequences of >500 residues). In some cases, PSI-BLAST returns multiple short aligned regions, none of which is long enough to pass the above requirements. In these cases, the failing hits are realigned to an HMM built from the seed, followed by alignment analysis. The seed and any accepted sequences are defined as a cluster and removed from *Q *(but not *E*). A new seed is then chosen from *Q *and the process is iterated until *Q *is empty.

This procedure results in a set of clusters, some of which may contain the same sequence(s). At this stage we merge compatible overlapping clusters. We rank all pairs of clusters by the number of sequences they have in common and attempt to merge pairs in order. For each pair, we choose the alignment with the greater number of aligned columns and designate this as cluster *A*; the other cluster becomes *B*. We build an HMM from the cluster *A *alignment and use the COACH algorithm [56] to align the entire cluster *B *alignment to this HMM (and therefore to cluster *A*). If the total fraction of gap characters in the merged alignment (that is, no. of gaps/(no. of sequences × no. of columns)) is less than 20%, and the mean percent identity is greater than 20%, the merge is accepted. Otherwise, if the alignment fails based on gap content alone, we trim columns with >30% gaps from the amino and carboxyl termini and reassess: the merge is accepted if cluster sequences align at least 70% of their residues, on average, within the trimmed 'core' alignment and if the core alignment contains >50% of the columns from the merged alignment and <10% gaps. Note, this procedure is computationally efficient, but can produce clusters that fail to meet the criteria set for global homology group books. In these cases, books are flagged for additional automated analysis and manual inspection in order to maintain quality control.

GHGCluster was used to cluster the human genome in the construction of the PhyloFacts Animal Proteome Explorer, including homologs from NR, using three iterations of PSI-BLAST.

#### Note on clustering splice and allelic variants

Our atomic unit in PhyloFacts clustering is the protein sequence, independent of its origin. Consequently, splice and allelic variants of a gene are not handled differently to genes from entirely different species during clustering, alignment and tree construction (although they would be interpreted differently during a subsequent phylogenomic analysis using the resource). If the variant retains the same domain structure and is globally alignable to other isoforms, it will be included in the global homology group cluster for those genes, otherwise it may end up in a different book in the resource. It should be noted that the presence of different isoforms for a gene can cause difficulties in phylogenomic inference if their common genome locus is not evident. Future releases of PhyloFacts will display this information and create links between different gene isoforms present in different books.

### Multiple sequence alignment

The alignment method is selected based on the type of book. For alignments of global homology group proteins, we normally use the MUSCLE software [57] to realign sequences obtained in clustering; in some cases, we use the SATCHMO software [58]. Both methods have outstanding performance evaluated on benchmark datasets. Alignments of structural domains are taken directly from the clustering algorithm (PSI-BLAST or T2K). The method used is indicated in the 'Book Details' section at the bottom of each book page, under 'Build method notes'. Multiple sequence alignments for all books (except those constructed to model solved three-dimensional structures) are masked to remove columns with >70% gaps prior to phylogenetic tree construction. Alignments are available for the family as a whole and for each subfamily; these can be downloaded or viewed using the Java-based Jalview software [59]. An annotated alignment, indicating SCI-PHY subfamily membership, is also available for viewing and download. Alignment statistics are provided, including average, minimum and maximum percent identity, fraction of gap characters in the MSA, and other relevant measurements.

### Defining book type

To be defined as a 'Global Homology' book, a multiple sequence alignment must meet the following criteria: first, ≤15% gap characters over the multiple sequence alignment; second, ≤30% columns with BLOSUM62 [60] sum-of-pairs scores < 0; third, difference between the longest and the shortest sequence in the alignment <150 amino acids; and fourth, all sequences align over ≥75% of their length. Books of type 'Domain' were required to match a structural domain (as determined by SCOP) or to correspond to a PDB structure. Books labeled as 'Conserved Region' required global-local alignment of sequences to the HMM (generally matching over 70% of the HMM match states). Most books labeled as 'Pending' are those that were produced by the GHGCluster program, but which failed the stringent 'Global Homology Group' alignment quality control tests; the final classification of these books to the different structural types is in progress.

### Subfamily identification

Subfamily identification is provided using the SCI-PHY (Subfamily Classification In Phylogenomics) software [61]. SCI-PHY is an automatic subfamily identification algorithm; given an input MSA, SCI-PHY uses Dirichlet mixture densities [62] and relative entropy to construct a hierarchical tree, and cuts the tree into subtrees to identify subfamilies using minimum-description-length principles. Extensive studies show SCI-PHY subfamilies correspond closely to both expert-identified subtypes and to conserved clades in phylogenetic trees (unpublished data, Brown DP, Krishnamurthy N, Sjölander K). The SCI-PHY server is available online [63].

### HMM construction for the family and individual subfamilies

The UCSC Sequence Alignment and Modeling (SAM) software is used to construct HMMs and in scoring sequences against HMMs [64]. This software was selected based on its outstanding performance in remote homology detection [17,64]. Family HMMs are constructed using the UCSC SAM w0.5 software. Subfamily HMMs (SHMMs) are constructed as described in [65]. Validation experiments on over 500 unique SCOP folds comparing subfamily and family HMMs show SHMMs to have high specificity in detecting functionally similar sequences and to improve the range of homolog detection with significant scores (unpublished data, Brown DP, Krishnamurthy N, Sjölander K).

### Protein structure and domain prediction

PFAM domains are identified using the consensus sequence for the family as a query using the PFAM gathering threshold as a cutoff. Matches to PDB structures are predicted by BLAST analysis of the family consensus sequence, using an E-value cutoff of 0.001 (that is, protein structure prediction based on inferred homology). Any putative homologous structures were aligned to the family HMM using local-local alignment (SAM parameter - sw 2). Transmembrane domains and signal peptides are predicted using the PHOBIUS server [29], selected due to its ability to differentiate between signal peptides and transmembrane domains.

### Phylogenetic tree construction and visualization

Because many of the protein superfamilies in the PhyloFacts resource span extremes of evolutionary divergence (for example, with pairwise identities <20%), tree topologies produced by different methods can often disagree. For this reason, most of the protein families in the resource contain several phylogenetic trees built using different algorithms, enabling biologists interested in these families to examine the differences and commonalities between the trees.

Neighbor-Joining trees are constructed using the PHYLIP software [66], using the default parameters for 'protdist' and 'neighbor' (JTT model [67] and no variation of rates). Maximum Likelihood trees are estimated using the PHYML software [68], also using the JTT model, four substitution rate categories, and a gamma-distributed model of rates (gamma = 1), and are set to optimize tree topology only. Maximum Parsimony trees are estimated using the PAUP* software [69], by taking an extended majority rule consensus of the most parsimonious trees obtained via ten repetitions of heuristic tree search. All trees are rooted using the midpoint method. As of 4 July 2006, of the 9,707 books in the library, 8,511 have at least one true phylogenetic tree constructed, and 3,613 have NJ, ML and MP trees. All books have had SCI-PHY subfamily analyses completed, and will eventually include Neighbor-Joining (including bootstrap values), Maximum Likelihood and Maximum Parsimony trees.

**Figure 4:**
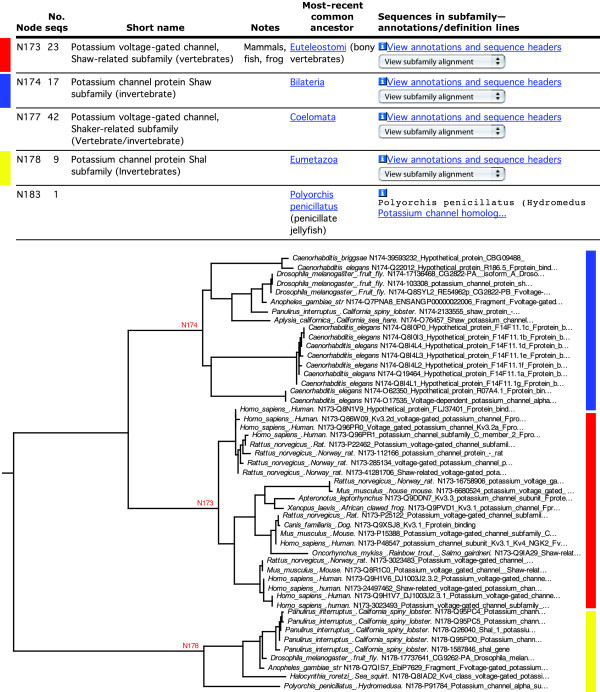
SCI-PHY subfamilies correspond closely to conserved phylogenetic clades. Shown here is the Maximum Likelihood (ML) tree and SCI-PHY subfamilies for the PhyloFacts book 'Voltage-gated K+ channels, Shaker/Shaw subtypes'. A branch of the ML tree is displayed, labeled with the corresponding SCI-PHY subfamilies. Subtrees containing sequences from a single subfamily are colored to show the correspondence between the SCI-PHY subfamilies and the ML tree.

Phylogenetic trees are displayed using ATV, a Java-based tree viewer [70]. Users can view any of the standard trees pre-estimated for the family or subtrees corresponding to SCI-PHY subfamilies. Phylogenetic trees can also be downloaded in NHX format. To facilitate a comparison with SCI-PHY subfamilies, the nodes in the phylogenetic trees containing sequences from a single SCI-PHY subfamily are annotated with the SCI-PHY subfamily number and annotation (Figure 4).

### Predicted critical residues

**Figure 5:**
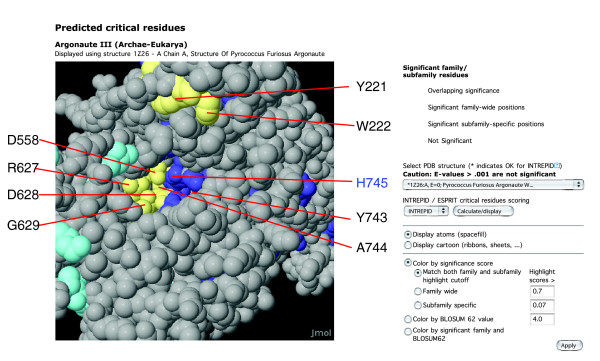
Key residue prediction using SCI-PHY subfamily-specific and family-wide conservation patterns. Shown above is the PDB structure for the *Pyrococcus furiosus *Argonaute protein (PDB structure 1Z26A), from the PhyloFacts book Argonaute III (Archaea-Eukarya). The structure has been colored to predict functional residues. Residues colored yellow are conserved within both subfamilies and across the family as a whole. Positions conserved only within individual subfamilies but not across the family are colored dark blue. Positions having sufficient conservation across the family, but potentially variable within one or more subfamilies are colored light blue. These conservation patterns are predicted for each book in the PhyloFacts resource; where homologous PDB structures can be identified, these patterns are plotted on the structure. Users can modify cutoffs for determining significance using the boxes at right. Most of the residues highlighted automatically by our conservation analyses, based on the default cutoffs, have been determined experimentally to be part of the active site [80-82] (labeled manually for this figure): R627, D628, G629, D558, Y743, H745). Y221 and W222 represent a prediction by this server. Structure viewing and interaction is enabled by the Jmol software.

Residues appearing to be important based on analysis of conservation patterns across the family as a whole, or within SCI-PHY subfamilies, are displayed under the header 'Predicted critical residues'. Key functional residues for the family as a whole are determined by multiple sequence alignment analysis. For each column *c*, we compute the log-odds of the positional conservation and the background conservation in the MSA: log (*F*_*c*_*/F*). Here, *F*_*c *_is the frequency of the most frequent amino acid at column *c*, and *F *is the average value of *F*_*c *_over the multiple alignment. Subfamily-defining positions are computed similarly, based on a cut of the MSA into subfamilies using the SCI-PHY algorithm, and then averaging log-odds values across the subfamilies at each position. Positive log-odds values indicate higher-than-average conservation at a position, whereas log-odds values below zero reflect conservation that is lower than average; the magnitude of the log-odds gives a measure of the significance of the result. Computing the log-odds instead of the conservation *per se *enables us to differentiate truly informative positions from those that only appear conserved due to limited sequence divergence in the multiple alignment. Our default cutoffs for coloring residues based on this analysis are 0.7 for family conservation and 0.07 for subfamily conservation; cutoffs can be adjusted by the user. Conservation patterns are plotted on protein three-dimensional structures for the family using an interactive Java-based structure viewer, Jmol (Figure 5) [71].

### Novel sequence classification

Classification to a protein family is enabled by HMM scoring. Since HMM scoring is computationally intensive and the PhyloFacts resource contains almost 10,000 books (each containing a family HMM and potentially dozens of subfamily HMMs), we provide heuristic approaches enabling rapid classification of user-submitted sequences. For computational efficiency, we select books for HMM scoring via BLAST analysis of the submitted query against a dataset of over 2.5 million PhyloFacts training sequences using an E-value cutoff of 10; this significantly reduces the number of HMM scores required without affecting sensitivity. Users can override this 'BLAST pre-screen' protocol using the 'Advanced' settings page. Books retrieved based on either protocol can then be selected for scoring the submitted sequence(s) against subfamily HMMs for additional specificity of functional classification.

## Future work

In future releases of the PhyloFacts resource we plan to include automated predictions of protein function using the SIFTER software. SIFTER will be used to provide predicted molecular functions and participation in biological processes for SCI-PHY subfamilies as well as for conserved evolutionary clades. Links between proteins, or between books, will be provided, to reflect the many types of relationships (for example, participating in the same pathway or complex, sharing a common domain or a predicted common ancestor, splice variation, and so on). Users will be able to navigate between proteins in the same pathway using the Cytoscape software, in which links to PhyloFacts books will be embedded. Users will be able to retrieve comparative (homology) models for selected proteins from the ModBase resource [72] through hyperlinks on book pages. Literature will be retrieved automatically for sequences in a book, and natural language processing software will be used to summarize the key points. We will expand the resource for improved coverage of key (animal) model organisms (human, mouse, *C. elegans*, *D. melanogaster*), and to keep our protein structure prediction library current. We plan to reduce redundancy in the library by combining books with significant sequence overlap. We will develop software tools to identify and include new family members as well as new experimental data (for example, the availability of solved structures, results from site-directed mutagenesis, protein-protein interactions, and so on). We will also include extensions to the subfamily HMM scoring protocol to differentiate between sequences representing a novel subtype and those that can be classified to the top-scoring subfamily (based on logistic regression analysis). Phylogenetic trees for each family will be extended to include strict consensus trees across two or more methods, and bootstrap analysis will be provided for Neighbor Joining trees. Finally, we plan to provide community annotation tools to enable biologists to upload their data, commentaries and hyperlinks to experimental data for members of protein families.

## Challenges to phylogenomic inference

Phylogenomic analysis of protein function is known to improve the accuracy of functional annotation, but has had restricted application due to its technical complexity. The PhyloFacts resource enables biologists who may have limited bioinformatics expertise to take advantage of pre-computed phylogenomic analyses for hundreds of thousands of proteins. New sequences can be classified to families and subfamilies using over 700,000 hidden Markov models, for increased functional specificity. The resource as a whole brings together many different types of bioinformatics analyses and data; the integration of these data and improved orthology determination enables biologists to avoid making new annotation errors at the outset, and to detect, and possibly correct, existing annotation errors.

Phylogenetic uncertainty and ambiguity remain significant challenges to phylogenomic inference of molecular function. Consensus analysis can be used to detect clades with support across two or more tree methods, but this approach could inadvertently be misleading if inherent biases in the methods are not taken into account. It is critical that the computational biology community and the systematics community work together to develop methods to assess the expected accuracy of phylogenetic methods for protein superfamily reconstruction; new simulation studies should be developed that model the kinds of structural and functional changes observed in protein superfamily evolution.

Other challenges to automating functional annotation through phylogenomics methods include: the lack of a standardized nomenclature for gene names across different model organisms, although the GO consortium efforts are making progress in this respect; natural structural and sequence divergence among family members causing difficulties in clustering; ensuring multiple sequence alignment accuracy when divergently related sequences are included in a cluster; and the persistence of database annotation errors. Database annotation errors should be correctable at the source, so that the primary sequence repositories (GenBank, UniProt, and so on) can be kept current.

One of the fundamental questions in phylogenomic inference of protein function is determining the evolutionary distance within which annotations may be transferred. Given the paucity of sequences with experimentally determined function, if annotations can only be transferred between orthologs (and are also restricted to annotations having experimental support), the vast majority of unknown sequences will remain without predicted function. Is this necessary or overkill? Analysis of different types of 'function' associated with proteins show that some types of attributes (for example, catalytic activity) persist over large evolutionary distances, while in other cases (for example, substrate specificity), functions can diverge extremely rapidly. Moreover, the degree to which different types of function persist over evolutionary distance can vary from one family to another. One intriguing possibility for the next generation of phylogenomic inference methods involves identifying attribute-specific evolutionary distances over which attributes may percolate.

Finally, assessing annotation accuracy is a very labor-intensive practice. Biological curators can spend days analyzing and annotating a single gene; to do this in high-throughput for thousands of sequences is clearly not feasible. An additional complication is that definitions of molecular 'function' or 'subfunction' are not at all standardized within biology. Instead, some biologists use the term 'function' very specifically (see for example, [3]) while others may use the term more loosely. Assessing annotation accuracy, and comparing the relative effectiveness of different function prediction protocols, also requires judgment calls regarding definitions of correctness. An annotation may be technically correct, but at such a high level that it is minimally helpful. For instance, a novel gene may be labeled as 'putative membrane protein', 'putative GPCR' and 'putative chemokine receptor'. If experimental studies show the protein to be a chemokine receptor, then only the third annotation would be particularly helpful to biologists, although all annotations would be technically correct. In other cases, an annotation may be technically incorrect, although quite close, due to annotation transfer from a paralog with a slightly different functional specificity (for example, Serotonin receptor type 1 versus Serotonin receptor type 2). Critically, there is also no community-accepted benchmark dataset or scoring function to evaluate methods of protein functional classification. These need to be developed to enable computational biologists to determine what types of inference methods are robust under what conditions, and where our methods fail. Efforts to develop true *de novo *function prediction efforts, analogous to the biennial CASP protein structure prediction experiments, are underway [73], and are likely to play an important role in improving our understanding of method accuracy in this important area.

## Additional data files

The following additional data files are available with the online version of this paper. Additional data file 1 provides a brief comparison of the Panther resource with PhyloFacts. Additional data file 2 is an illustration of detection of potential annotation errors using PhyloFacts analyses.

## Supplementary Material

Additional data file 1 Brief comparison of the Panther resource with PhyloFacts Click here for file

Additional data file 2 Illustration of detection of potential annotation errors using PhyloFacts analyses Click here for file

## Figures and Tables

**Table 1:** Distribution of various book types in PhyloFacts

Book type	No. of books in PhyloFacts
Global homology group	2,567
Domain	5,363
Conserved region	72
Motif	29
Pending	1,679

PhyloFacts contains books of different structural types. Global homology group: sequences sharing the same domain architecture, aligned globally. Domain: sequences sharing a common structural domain (defined experimentally), aligned only along that domain. Conserved regions: sequences sharing a common region with no obvious homology to a solved structure, aligned along that region. Motifs: highly conserved amino acid signatures typically <50 amino acids. Pending: all other books, including clusters produced by GHGCluster that did not pass the global homology group criteria (and in the process of being evaluated for classification to one of the three main categories). Results reported as of 11 July 2006.

**Table 2:** Comparison of PhyloFacts with other functional classification resources

	PhyloFacts	Panther	TIGRFAMs	Sanger PFAM	SMART	InterPro	Superfamily
**Analysis of user-submitted sequences**							
Classification to full-length protein families	Yes	No*	Yes				
Subfamily level classification	Yes	Yes	Yes				
Domain level classification	Yes		Yes	Yes	Yes	Yes	Yes
DNA sequence analysis	Yes			Yes		Yes	Yes
Batch-mode sequence inputs allowed	Yes			Yes	Yes		Yes
**Analysis required for phylogenomic inference**							
Clusters based on full-length protein families	Yes	No*	Yes				
Phylogenetic trees for full-length protein families	Yes						
Subfamily identification	Yes	Yes	Yes			Partial^†^	
GO data for individual sequences	Yes	No^‡^				Yes	
GO data for clusters	Yes	No^‡^	Yes	Yes	Yes	Yes	
GO evidence codes	Yes					Yes	
EC numbers for individual sequences	Yes	Yes	Yes				
EC numbers for each cluster	Yes		Yes				
Taxonomy information	Yes	Yes	Yes	Yes	Yes	Yes	Yes
**Analyses required for function inference based on structure**							
Phylogenetic trees for single domains	Yes			Yes			
Clusters based on domains	Yes		Yes	Yes	Yes	Yes	Yes
Predicted three-dimensional structure for a protein family	Yes			Yes	Yes	Yes	Yes
Predicted critical residues	Yes						
PDB structure visualization	Yes			Yes			
PFAM domains	Yes		Yes	Yes	Yes	Yes	
Transmembrane domain prediction	Yes				Yes	Yes	
Signal peptide prediction	Yes				Yes	Yes	
SCOP classification	Yes			Yes	Yes	Yes	Yes
Links to PDB	Yes			Yes	Yes	Yes	
**Additional protein family data**							
Retrieval of relevant literature for individual families	Partial	Yes	Yes	Yes	Yes	Yes	
Extended protein family annotation		Yes		Yes	Yes	Yes	
Clusters of interacting domain families				Yes			
Graphic displays of related domain architectures				Yes	Yes	Yes	Yes

This table compares the functionalities provided by PhyloFacts with those of standard functional classification resources for structural phylogenomic analysis. PhyloFacts is the only online resource that enables structural phylogenomic inference of protein function, including clustering of sequences into structural equivalence classes (that is, containing the same domain architecture), construction of phylogenetic trees, identification of functional subfamilies, subfamily hidden Markov models and structure prediction. This differentiates PhyloFacts from other resources that almost exclusively enable domain prediction (for example PFAM, Superfamily) and those such as TIGRFAMs that cluster full-length protein sequences but do not integrate structural and phylogenomic analysis. Reported as of May 2006. *Although Panther asserts that its families contain globally alignable sequences, this is not always the case (see additional data file 1 for details). ^†^InterPro has defined parent/child relationships between some entries that are considered equivalent of family/subfamily relationships. But these are not defined for every cluster. ^‡^Panther provides its own ontology terms instead of the standard GO annotations. Links to the resources used for this comparison: PhyloFacts Resource [11]; Celera Genomics Panther Classification [74]; TIGRFAMs [75]; PFAM HMM library at the Sanger Institute [76]; SMART [77]; InterPro [78]; Superfamily [79].

**Table 3:** Fractional coverage of genomes

Model organism	Number of sequences	Fractional coverage
*Homo sapiens*	27,960	0.82
*Escerichia coli*	4,237	0.70
*Arabidopsis thaliana*	26,207	0.75
*Caenorhabditis elegans*	26,032	0.64
*Drosophila melanogaster*	19,178	0.74

The fraction of sequences from different model organisms that can be functionally classified by PhyloFacts to one of the books in the resource, based on BLAST search against PhyloFacts training sequences, using an E-value cutoff of 0.001.
