# Supplementary material for: PhyloFacts: an online structural phylogenomic encyclopedia for protein functional and structural classification
Source: Genome Biol. 2006 Sep 14;7(9):R83. doi: 10.1186/gb-2006-7-9-r83 (PMC1794543; doi:10.1186/gb-2006-7-9-r83)
Supplement: Additional data file 1 — Brief comparison of the Panther resource with PhyloFacts [file gb-2006-7-9-r83-S1.pdf]

Comparing the Berkeley Phylogenomics Group PhyloFacts resource and the Celera Genomics Panther Classification System

Although PhyloFacts and Panther (http://www.pantherdb.org/) have a common origin (KS was Principal Scientist of the Protein Informatics Group that developed the Panther tools at Celera Genomics), and both provide subfamily HMMs for classification of sequences to functional subtypes, there are several distinguishing features between the two systems. Of the two resources, only PhyloFacts includes phylogenetic trees estimated using standard tools (e.g., Neighbor-Joining, Maximum Parsimony and Maximum Likelihood), GO annotations and evidence codes, predicted 3D structures and critical residues, and other analyses. Perhaps most critically, PhyloFacts books are labelled with the book type, i.e., whether the cluster corresponds to globally alignable proteins (global homology groups), to structural domains, conserved regions or motifs. This enables biologists to interpret assignments of sequences to each book appropriately.

Although Panther asserts that its families contain globally alignable sequences, this is not always the case. For instance, the Panther Leucine-Rich Transmembrane Proteins [PTHR23154] family contains human Slit homolog 3 [UniProt: SLIT3_HUMAN], human Toll-like receptor 5 [UniProt: TLR5_HUMAN], and dog bone/cartilage proteoglycan I [UniProt: PGS1_CANFA]. A brief review of the three proteins reveals extreme differences in length (369aa for the dog proteoglycan protein, 858aa for the human toll-like receptor and 1,523aa for human Slit homolog 3), and very different domain structures. Nevertheless, they are classified into a single Panther family based on agreement along a single region (the leucine-rich repeat region). Moreover, the dog bone/cartilage proteoglycan I protein has no detectable transmembrane domain.

By contrast, these three sequences are classified in the PhyloFacts resource to separate global homology groups. SLIT3_HUMAN (sp|O75094) is found in several books, only one of which (SLIT; bpg000558) is of type *Global Homology*, indicating sequences in the book share a common domain structure. (The other books containing SLIT3_HUMAN are either of type *Other* (pending classification) or of type *Domain*, indicating local homology only.) Similarly, TLR5_HUMAN (sp|O60602) is found in several books, but only one (TLR5; bpg000142) is of type *Global Homology*. PGS1_CANFA (sp|O02678) is found in several books based on local similarity, and only one (SLRP (Small Leucine-Rich Proteoglycan; bpg005007) is of type *Global Homology*.
