# Supplementary material for: PhyloFacts: an online structural phylogenomic encyclopedia for protein functional and structural classification
Source: Genome Biol. 2006 Sep 14;7(9):R83. doi: 10.1186/gb-2006-7-9-r83 (PMC1794543; doi:10.1186/gb-2006-7-9-r83)
Supplement: Additional data file 2 — Illustration of detection of potential annotation errors using PhyloFacts analyses [file gb-2006-7-9-r83-S2.pdf]

Phylogenomic analysis detects misannotation of a putative lamprey odorant receptor

PhyloFacts integrates a wide array of biological data and informatics methods for protein families, organized on the basis of structural similarity and by evolutionary relationships. This enables a biologist to examine a rich array of experimental data and bioinformatics predictions for a protein family, and to quickly and accurately infer the function of a protein in an evolutionary context.

Here we present an illustration where the PhyloFacts resource enables us to detect a potential error in database annotation by analyzing a cluster of homologs as a unit; database annotation errors tend to stand out as anomalous against a backdrop of otherwise consistent annotations.

G-protein coupled receptor (GPCR) classification is notoriously difficult, with many receptors having no known ligand (termed "orphan receptors"). One such orphan, a GPCR from river lamprey [UniProt: Q9YHY4], is annotated as "Putative odorant receptor LOR3," based on its expression in the olfactory epithelium [1]. Standard profile/HMM-based analyses (e.g., PFAM, SMART and the NCBI CDD) only match this protein to the PFAM 7TM_1 class, containing dozens of subtypes. BLAST analysis shows other putative odorant receptors from river lamprey (submitted by the same authors) as top hits, followed by trace amine receptors (Figure 1). However, analyses of Phylofacts data shows this sequence to be related to the biogenic amine receptors, being closest to trace amine receptors in particular.

The lamprey protein can be found in four "books" in the PhyloFacts resource, each containing clusters of various GPCRs (Figure 2). Analysis of phylogenetic trees in these books show this sequence (and the other putative odorant receptors detected by BLAST) to be located within subtrees containing trace amine receptors (Figures 3 & 4) (see PhyloFacts books bpg004950, bpg000525 and bpg000543) and to be quite different from experimentally confirmed odorant receptors .

The figures in the following pages include:

Figure 1: BLAST Analysis of Q9YHY4

Figure 2: PhyloFacts search results for Q9YHY4

Figure 3: Maximum Likelihood tree from the "Global homology" book GPCR: Aminergic (multiple subtypes); bpg004950)

Figure 4: Parsimony tree from the "Global homology" book GPCR: Aminergic (multiple subtypes); bpg004950)

1. Berghard A, Dryer L: A novel family of ancient vertebrate odorant receptors. J Neurobiol 1998, 37(3):383-392.

Sequences producing significant alignments:		Score (Bits)	E Value
gb|AAC82381.1|	putative odorant receptor LOR3 [Lampetra fluviati	630	3e-179
gb|AAC82382.1|	putative odorant receptor LOR4 [Lampetra fluviati	227	7e-58
emb|CAF93600.1|	unnamed protein product [Tetraodon nigroviridis]	206	1e-51
ref|NP_001010827.1|	trace amine associated receptor 7b [Mus m...	201	5e-50
ref|NP_001010831.1|	trace amine-associated receptor 9 [Mus mu...	201	5e-50
gb|AAK71254.1|	trace amine receptor 15 [Rattus norvegicus] >s...	200	9e-50
gb|AAK71240.1|	trace amine receptor 3 [Homo sapiens] >gb|AAO2...	199	1e-49
gb|AAK71241.1|	trace amine receptor 3 [Rattus norvegicus] >re...	199	2e-49

Figure 1. BLAST Analysis of Q9YHY4 annotated as "Putative Odorant receptor" from Lamprey. The most significant BLAST hits to Q9YHY4 when searched against the NR database. The two top hits are annotated putative odorant receptor from Lamprey. However the majority of the following hits are annotated as Trace Amine receptors.

BPG books - matches to words in "Q9YHY4"

Short name	Type	Pfam	Notes
GPCR: Rhodopsin-like VI	Other	7tm_1	This book contains sequences from various Class-A Rhodopsin-like GPCRs. Homologs are found throughout animals.
GPCR: Amine and Peptide receptors.	Other	7tm_1	This book contains GPCRs for amines (e.g., dopamine, serotonin, trace amine, and similar ligands) and peptides, (e.g., somatostatin, cholecystokinin, neuropeptide Y and others). Homologs are found throughout the animal kingdom.
7TM receptors (multiple subtypes)	Pending	7tm_1	This family spans a wide number of subtypes, including aminergic, chemokine and related receptors.
GPCR: Aminergic (multiple subtypes)	Global homology	7tm_1	

Figure 2. Search results for Q9YHY4 in the Phylofacts database returns four books one of which is of type Global Homology (GPCR: Aminergic (multiple subtypes); *bpg004950*).

A phylogenetic analysis of the lamprey proteins from these books shows that in all the books, these proteins are found nested within biogenic amine receptors and most closely related to Trace amine receptors in particular. The illustration presented below shows Maximum Likelihood and Parsimony trees from the book *GPCR: Aminergic (multiple subtypes) (bpg004950).*

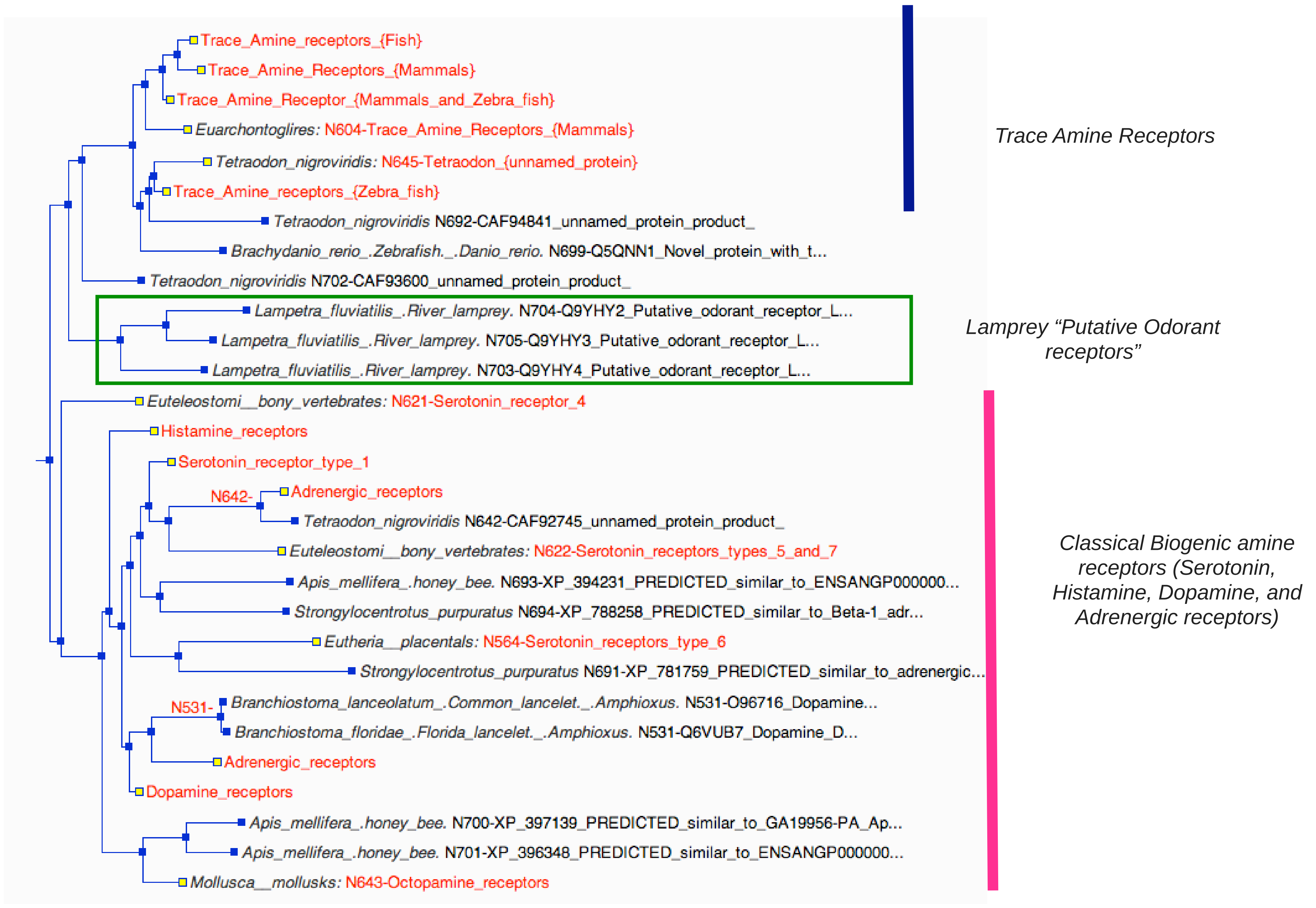

Figure 3. Maximum Likelihood tree from the "Global homology" book *GPCR: Aminergic (multiple subtypes); (bpg004950)*. The nodes with consistent annotation of proteins have been collapsed and labelled appropriately (in red). The lamprey proteins annotated as "Putative odorant receptors" are boxed in green. The subtree containing Trace amine receptors is highlighted with a blue line and the subtree containing the classical biogenic amine receptors is highlighted with a pink line.

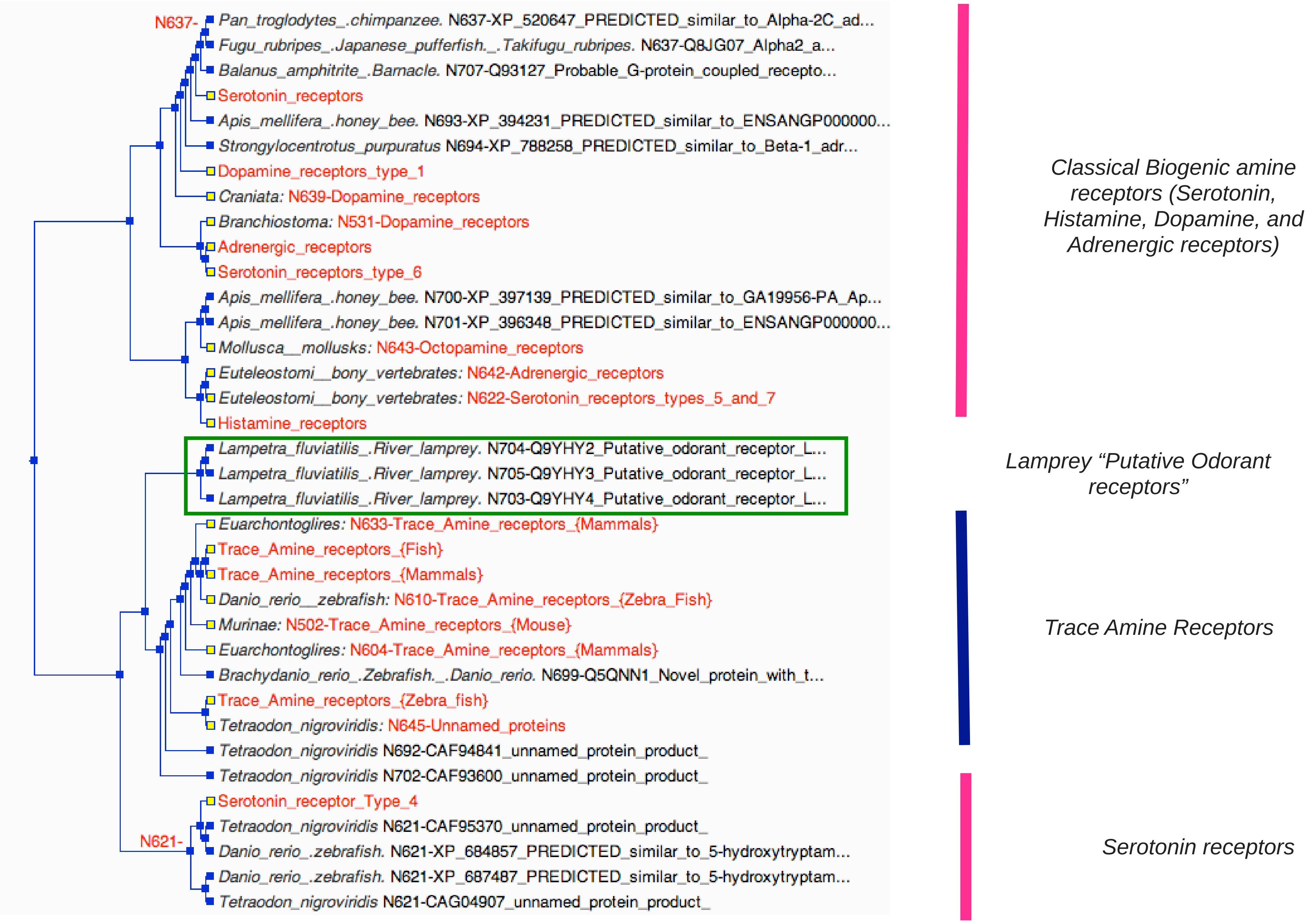

Figure 4*.* Parsimony tree from the “Global homology” book *GPCR: Aminergic (multiple subtypes); bpg004950)*. The nodes with consistent annotation of proteins have been collapsed and labelled appropriately (in red). The lamprey proteins annotated as “Putative odorant receptors” are boxed in green. The subtree containing Trace amine receptors is highlighted with a blue line and the subtree containing the classical biogenic amine receptors is highlighted with a pink line.
